# Predictive impact of C-reactive protein to albumin ratio for recurrent or metastatic head and neck squamous cell carcinoma receiving nivolumab

**DOI:** 10.1038/s41598-021-82448-1

**Published:** 2021-02-02

**Authors:** Kenro Tanoue, Shingo Tamura, Hitoshi Kusaba, Yudai Shinohara, Mamoru Ito, Kenji Tsuchihashi, Tsuyoshi Shirakawa, Taiga Otsuka, Hirofumi Ohmura, Taichi Isobe, Hiroshi Ariyama, Sakuya Koreishi, Yuzo Matsushita, Hozumi Shimokawa, Risa Tanaka, Kenji Mitsugi, Koichi Akashi, Eishi Baba

**Affiliations:** 1 Department of Medicine and Biosystemic Science, Graduate School of Medical Sciences, Kyushu University, 3-1-1 Maidashi, Higashi-ku, Fukuoka, 812-8582 Japan; 2 Department of Hematology, Oncology and Cardiovascular Medicine, Kyushu University Hospital, 3-1-1 Maidashi, Higashi-ku, Fukuoka, 812-8582 Japan; 3 Karatsu Higashi-Matsuura Medical Association Center, 2566-11 Chiyodamachi, Karatsu, 847-004 Japan; 4 Department of Internal Medicine, Minato Medical Clinic, 3-11-3-201 Nagahama, Chuou-ku, Fukuoka, 810-8539 Japan; 5 Department of Medical Oncology, Clinical Research Institute, National Hospital Organization Kyushu Medical Center, 1-8-1, Jigyouhama, Chuou-ku, Fukuoka 810-0065 Japan; 6 Department of Medical Oncology, Hamanomachi Hospital, 3-3-1 Nagahama, Chuou-ku, Fukuoka, Fukuoka 810-8539 Japan; 7 Department of Oncology and Social Medicine, Graduate School of Medical Sciences, Kyushu University, 3-1-1 Maidashi, Higashi-ku, Fukuoka, 812-8582 Japan

**Keywords:** Head and neck cancer, Tumour biomarkers, Tumour immunology

## Abstract

Although the neutrophil to lymphocyte ratio (NLR) was reported to be a predictive biomarker for clinical outcomes in various types of cancer, including recurrent or metastatic head and neck cancer (R/M HNSCC) treated with nivolumab, the usefulness of the pretreatment C-reactive protein/albumin ratio (CAR) as a prognostic marker remains to be clarified. This study aimed to analyze the clinical usability of the CAR in comparison with that of the NLR. 46 R/M HNSCC patients treated with nivolumab were retrospectively analyzed. The optimal cutoff value for the CAR was calculated using receiver operating characteristic curve analysis. The optimal cutoff value for the CAR was set to 0.30. On multivariate analyses, a high CAR was significantly associated with poor overall survival (adjusted HR, 2.19; 95% CI, 1.42–3.47; *p* < 0.01) and progression-free survival (adjusted HR, 1.98; 95% CI, 1.38–2.80; *p* < 0.01). The overall response rate and disease control rate for the high CAR patients were lower than for the low CAR patients. The CAR had significantly higher area under the curve values than the NLR at 2 and 4 months. The pretreatment CAR might be an independent marker for prognosis and efficacy in R/M HNSCC patients treated with nivolumab.

## Introduction

Each year, more than 835,000 newly diagnosed cases of head and neck squamous cell carcinoma (HNSCC) and approximately 431,000 deaths related to HNSCC are recorded worldwide^1^. Despite the development of multidisciplinary treatments, the prognosis of head and neck cancer has remained poor^2^.

Treatment strategies for recurrent or metastatic head and neck cancer (R/M HNSCC) are evolving rapidly. Nivolumab, a monoclonal antibody against programmed cell death 1 (PD-1) has been widely used as the standard treatment for R/M HNSCC, because, in the CheckMate 141 study, nivolumab significantly improved the overall survival (OS) of platinum-refractory R/M HNSCC compared to conventional therapy^3^. PD-1 is an immune checkpoint receptor that limits the effector functions of T cells. PD-1 inhibitors exert anti-tumor effects by blocking the interaction of PD-1 on T cells with the immune-suppressing ligand programmed cell death ligand 1 (PD-L1) on tumor cells. Currently, nivolumab has been used as the standard treatment in various types of cancer.

However, in the CheckMate 141 study, a high level of PD-L1 expression in the tumor did not reflect the anti-tumor effect of nivolumab^3^, even though PD-L1 was found to be expressed in 50–60% of HNSCCs^4^. Furthermore, only a limited population (approximately 20%) of cases responded to nivolumab treatment alone^3^. Therefore, determining the prognostic factors for nivolumab is one of the most important tasks, but there are still few reports of it. Huang et al. recently reported an association between a large immune response after immunotherapy and clinical response in melanoma, but higher systemic inflammation at baseline was also demonstrated to be closely correlated with poor clinical outcome, indicating that it was a very important factor in validating the prognostic value of immunotherapy^5^.

Recently, various biomarkers reflecting inflammation have been reported to be associated with cancer prognosis. Specifically in nivolumab-treated several cancers including R/M HNSCC, the neutrophil-to lymphocyte ratio (NLR) has attracted attention as a predictive biomarker^6–10^. On the other hand, the C-reactive protein (CRP) to albumin ratio (CAR), a new prognostic marker, has been reported as an independent prognostic factor in various tumors^11–17^. In patients with hypopharyngeal and laryngeal cancers after invasive surgery, the preoperative CAR was reported to be associated with OS and disease-free survival^18^.

Emerging evidence suggests that cancer-associated inflammation and nutritional status play a critical role in the progress of tumors^19^ and that these are also closely related to cancer-related cachexia^20^. Therefore, validating the inflammatory biomarker reflecting nutrition status such as the CAR, is of great significance and if it were a more accurate prognostic factor than the NLR, it could be clinically important because it could be easily calculated and used at the clinical site.

However, the prognostic usability of the pretreatment CAR in R/M HNSCC treated with nivolumab remains to be investigated. Furthermore, to the best of our knowledge, there are no reports comparing the prognostic usefulness of the pretreatment CAR with that of the pretreatment NLR in R/M HNSCC patients.

Therefore, the association between the pretreatment CAR and clinical outcomes was retrospectively investigated in R/M HNSCC patients treated with nivolumab. The prognostic utilities of the pretreatment CAR and NLR were also compared.

## Results

### Patients’ characteristics

A total of 46 R/M HNSCC patients (38 males, 8 females) were enrolled in this study (Table 1). The median age of the patients was 66 years (range 41–87 years). The numbers of patients with Eastern Cooperative Oncology Group (ECOG) performance status (PS) 0, 1, and 2 or worse were 10 (22%), 22 (48%), and 14 (30%), respectively. The primary tumor site was: oral cavity in 19 patients (41%); nasopharynx in 2 (4%); oropharynx in 9 (20%); hypopharynx in 11 (24%); larynx in 3 (7%); and external canal in 2 (4%). Well-, moderately-, and poorly-differentiated squamous cell carcinomas were observed in 15 (32%), 11 (24%), and 3 (7%) patients, respectively, whereas histological features for differentiation were unknown in the remaining 17 (37%) patients. In most cases, except for oropharyngeal carcinoma, p16 status was not measured, and there were only five p16-positive cases. Nivolumab was administered as the 1st-line therapy in 3 (7%), 2nd-line therapy in 29 (63%), and 3rd- or later-line therapy in 14 (30%) patients. The median pretreatment body mass index (BMI) (kg/m^2^), CRP (mg/dl), serum albumin (g/dl), CAR and NLR were 0.80, 3.70, 0.24 and 4.61, respectively. In terms of prior therapy, 44 (96%) patients had a history of platinum-containing therapies. Taxane-containing therapies were given to 19 (41%) patients. Twenty-four (52%) patients were treated with cetuximab-containing therapies. Radiation therapy was performed in 39 (85%) patients. Tumor surgery was performed in 29 (63%) patients. Patterns of disease status included locoregional recurrence in 23 (50%) patients, distant recurrence in 17 (37%) patients, and advanced without any history of curative therapy in 6 (13%) patients. Ten (22%) patients had arterial infiltration on computed tomography (CT) or magnetic resonance imaging (MRI). Thirty-three (72%) patients harbored a measurable lesion.

**Table 1:** Patients’ characteristics (n = 46).

Characteristics	Number (N = 46)	%
**Age (years)**		
Median (range)	66 (41–87)	
**Gender**		
Male	38	83
Female	8	17
**ECOG PS**		
0	10	22
1	22	48
2 ≦	14	30
**Primary tumor site**		
Oral cavity	19	41
Nasopharynx	2	4
Oropharynx	9	20
Hypopharynx	11	24
Larynx	3	7
External ear canal	2	4
**Histological differentiation**		
Well	15	32
Moderately	11	24
Poorly	3	7
Unknown	17	37
**p16 status**		
Positive	5	11
Negative	6	13
Unknown	35	76
**Nivolumab line**		
1	3	7
2	29	63
3≦	14	30
BMI (range) (kg/m^2^)	19 (12.9–26.8)	
CRP median (range) (mg/dL)	0.80 (0.01–14.97)	
Serum albumin median (range) (g/dL)	3.7 (2.5–4.5)	
CAR median (range)	0.24 (0.006–4.99)	
NLR median (range)	4.61 (1.07–20.4)	
**History of previous therapy**		
Platinum contained therapy	44	96
Taxane contained therapy	19	41
Cetuximab contained therapy	24	52
Radiation therapy	39	85
Tumor surgery	29	63
**Disease status**		
Locoregional recurrence	23	50
Distant recurrence	17	37
Advanced at diagnosis	6	13
**Distant metastases**		
Yes	27	59
No	19	41
**Arterial infiltration**		
Yes	10	22
No	36	78
**Measurable lesions**		
Yes	33	72
No	13	28

### Treatment response and survival outcome

**Figure 1:**
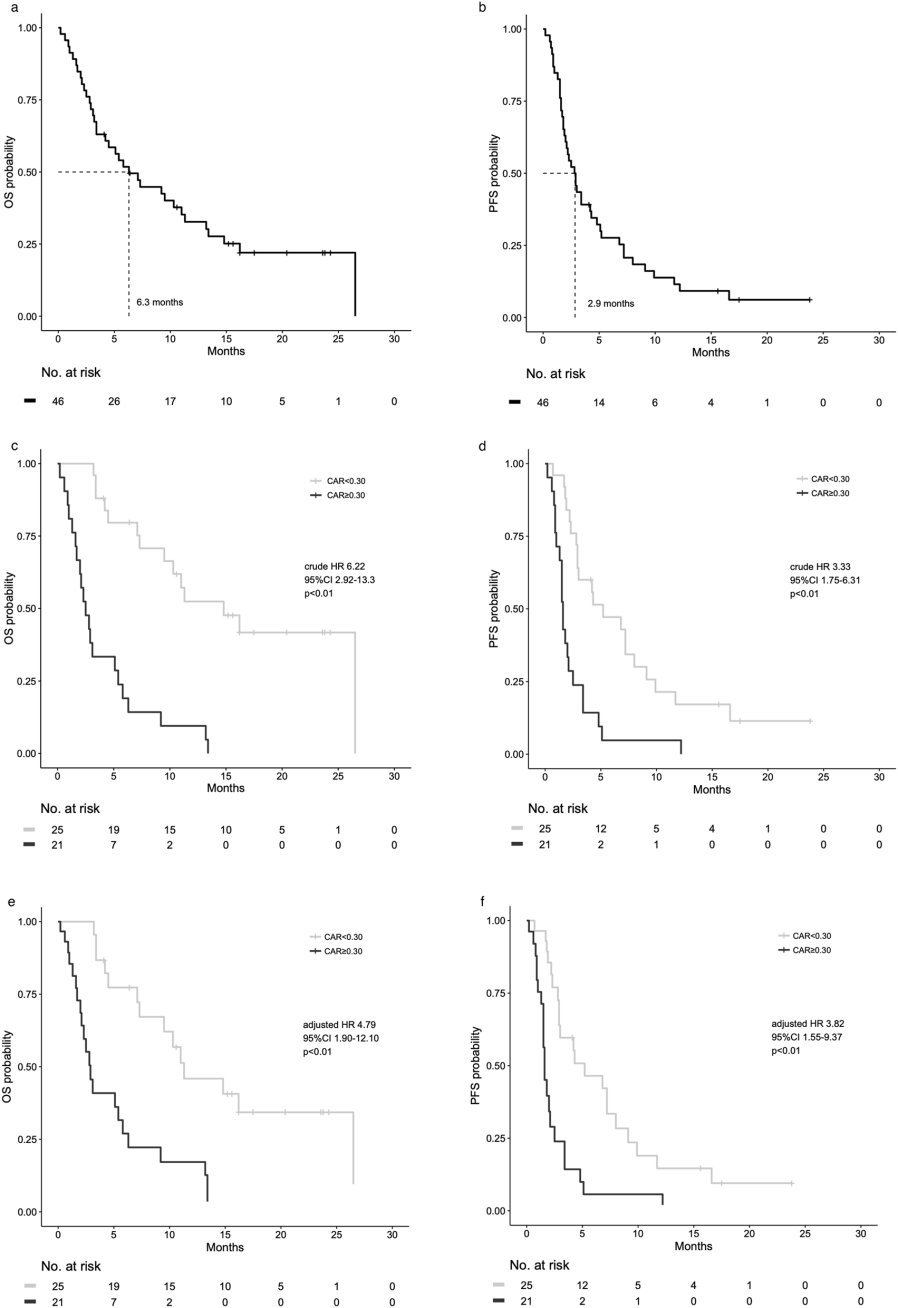
Kaplan–Meier curves of (**a**) overall survival in all patients and (**b**) progression-free survival in all patients. Kaplan–Meier curves of (**c**) crude overall survival and (**d**) crude progression-free survival according to the CAR. Black line indicates a high CAR (CAR ≥ 0.30), and the silver line indicates a low CAR (CAR < 0.30). Adjusted survival curves stratified by the CAR Cox curves for (**e**) overall survival adjusting for ECOG PS, arterial infiltration, and NLR, and (**f**) progression-free survival adjusting for ECOG PS, radiation history, and the NLR. ECOG PS: Eastern Cooperative Oncology Group performance status, CAR: C-reactive protein to albumin ratio, NLR: neutrophil to lymphocyte ratio.

Best overall response was found in 33 patients with measurable lesions (Table 2). Partial response (PR) was observed in 5 patients, and stable disease (SD) was observed in 9 patients. Among 46 patients, 35 deaths and 42 events for PFS occurred respectively. The median OS was 6.3 months (95% confidence interval (CI) 3.4–11 months), and the median progression free survival (PFS) was 2.9 months (95% CI 1.8–4.3 months) (Fig. 1a,b).

**Table 2:** Clinical outcomes of patients treated with nivolumab (n = 46).

		%
**Best overall responses of patients with measurable lesions**	N = 33	
PR	5	15
SD	9	27
PD	14	42
NE	5	15
ORR (%)		15
DCR (%)		42
**Best overall responses of patients without measurable lesions**	N = 13	
NonCR/nonPD	5	38
PD	5	38
NE	3	23

### Optimal cutoff level for the CAR

**Figure 2:**
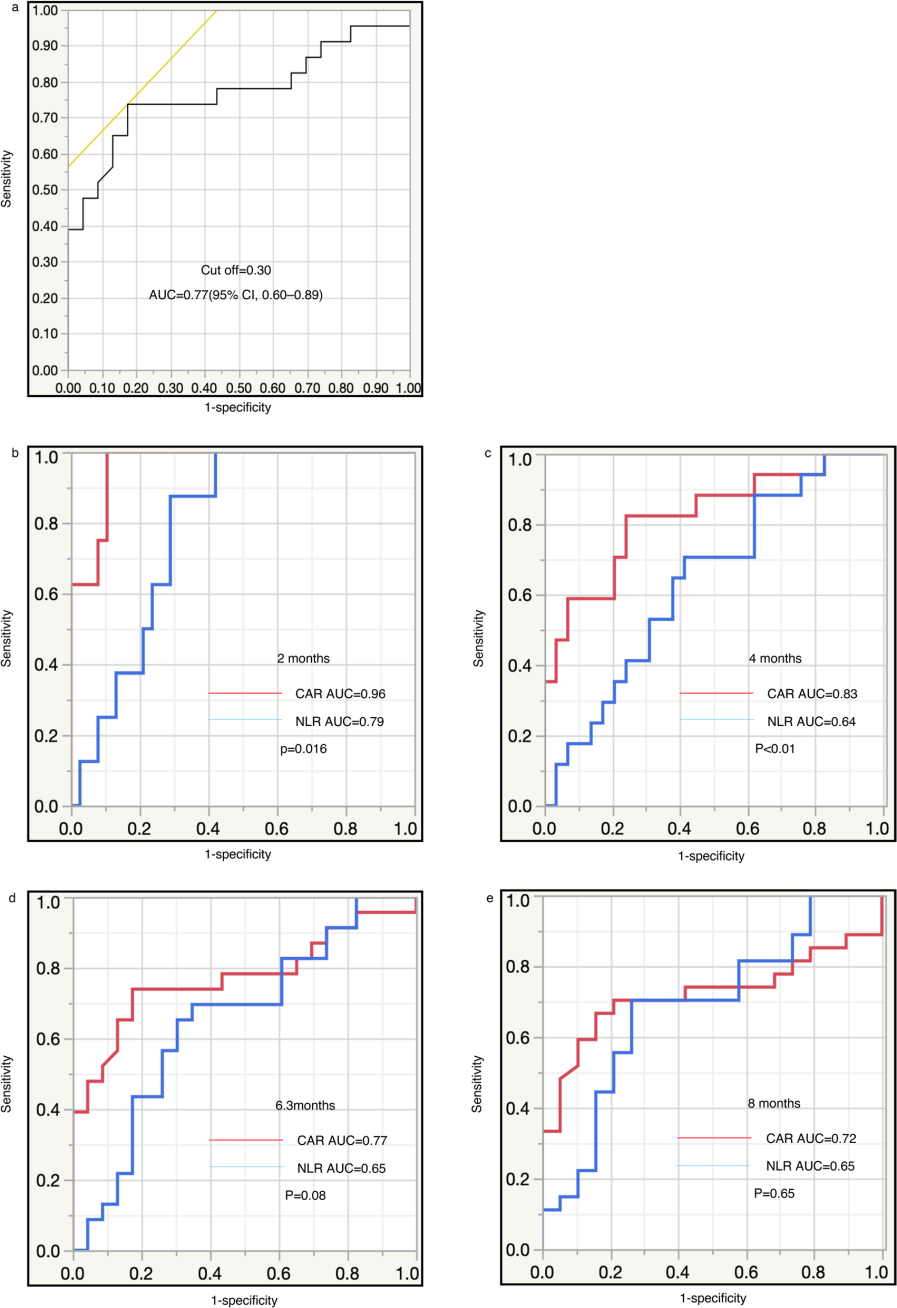
(**a**) Receiver operating characteristic curve to determine the cutoff value for the CAR level. Comparison of the areas under the receiver operating curves for outcome prediction between the two inflammation-based prognostic scores [CAR and NLR] at (**b**) 2 months, (**c**) 4 months, (**d**) 6.3 months, and (**e**) 8 months. CAR: C-reactive protein to albumin ratio, NLR: neutrophil to lymphocyte ratio.

The survival status (dead or alive) at 6.3 months, which was the median OS, was used as the state variable. The area under curve (AUC) was calculated as 0.77 (95% CI 0.60–0.89) for the CAR (Fig. 2a). The optimal cutoff value for the CAR was 0.30, which corresponded to the maximum sum of sensitivity and specificity on the receiver operating characteristic (ROC) curve.

### Impact of the CAR on clinical outcomes

The crude OS and PFS curves stratified by the cutoff of the CAR are shown in Fig. 1c,d. The OS of patients with a high CAR was significantly shorter than that of patients with a low CAR (median OS, 2.5 months and 14.8 months, respectively; crude hazard ratio (HR), 6.22; 95% CI, 2.92–13.3; *p* < 0.01) (Fig. 1c). Similarly, PFS in patients with a high CAR was significantly shorter than that in patients with a low CAR (median OS, 1.6 months and 5.2 months, respectively, crude HR, 3.33; 95% CI, 1.75–6.31; *p* < 0.01) (Fig. 1d).

### Adjusted survival analysis stratified by the CAR

On univariate analysis, ECOG PS (≧ 2 vs. 0–1, HR, 3.66; 95% CI, 1.77–7.59; *p* < 0.01), arterial infiltration (yes vs. no, HR, 4.47; 95% CI, 2.07–9.64; *p* < 0.01), the NLR (≧ 5 vs. < 5, HR, 2.30; 95% CI, 1.16–4.56; *p* = 0.02), and the CAR (continuous variable, HR, 2.58; 95% CI, 1.79–3.82; *p* < 0.01) were significantly associated with OS. When multivariate analysis was performed with these 4 factors, a high CAR was independently associated with OS (continuous variable, adjusted HR, 2.19; 95% CI, 1.42–2.47; *p* < 0.01) (Table 3).

**Table 3:** Univariate and multivariate analyses of OS and PFS for nivolumab-treated patients.

Variable	OS						PFS					
	Univariate analysis			Multivariate analysis			Univariate analysis			Multivariate analysis		
	HR	95%CI	*p* value	HR	95%CI	*p* value	HR	95%CI	*p* value	HR	95%CI	*p* value
Age (≥ 70 vs. < 70 year)	1.06	0.50–2.24	0.88				0.86	0.43–1.71	0.66			
Sex (Female vs. male)	1.92	0.83–4.43	0.13				1.21	0.54–2.76	0.64			
ECOG PS (≥ 2 vs. 0–1)	3.66	1.77–7.59	< 0.01	2.43	1.03–5.74	0.04	2.03	1.06–3.90	0.03	1.34	0.59–3.03	0.48
**Disease status**												
Locoregional recurrence	1						1					
Distant recurrence	1.23	0.59–2.57	0.58				0.97	0.50–1.89	0.93			
Advanced	1.44	0.52–4.00	0.49				1.19	0.47–2.99	0.72			
Chemotherapy line (≥ 3 vs. 1–2)	1.13	0.55–2.33	0.74				0.99	0.51–1.92	0.98			
Distant metastases (yes vs. no)	0.81	0.41–1.61	0.55				0.96	0.52–1.79	0.9			
Arterial infiltration (yes vs. no)	4.47	2.07–9.64	< 0.01	1.63	0.57–4.66	0.36	2.05	0.997–4.21	0.051			
Cmab history (yes vs. no)	0.86	0.44–1.70	0.67				1.21	0.66–2.23	0.54			
Radiation history (no vs. yes)	1.37	0.52–3.60	0.53				2.35	1.02–5.45	0.045	2.93	1.23–6.95	0.015
Tumor surgery history (yes vs. no)	1.19	0.58–2.45	0.64				1.27	0.67–2.40	0.46			
Platinum history (yes vs. no)	2.55	0.35–18.7	0.36				1.03	0.25–4.29	0.97			
NLR (≥ 5 vs. < 5)	2.3	1.16–4.56	0.018	0.86	0.33–2.21	0.75	2	1.08–3.69	0.027	1.24	0.55–2.80	0.60
CAR	2.58	1.79–3.82	< 0.01	2.19	1.42–3.47	< 0.01	2.01	1.47–2.72	< 0.01	1.98	1.38–2.80	< 0.01

In nivolumab-treated patients, ECOG PS (≧ 2 vs. 0–1, HR, 2.03; 95% CI 1.06–3.90; *p* = 0.03), radiation history (no vs. yes, HR, 2.35; 95% CI 1.02–5.45; *p* = 0.045), the NLR (≧ 5 vs. < 5, HR, 2.00; 95% CI, 1.08–3.69; *p* = 0.03) and the CAR (continuous variable, HR, 2.01; 95% CI, 1.47–2.72; *p* < 0.01) were significantly related to PFS on univariate analysis. On multivariate analysis of these 4 factors, radiotherapy history (no vs. yes, adjusted HR, 2.93; 95% CI, 1.23–6.95; *p* = 0.015) and a high CAR (continuous variable adjusted HR, 1.98; 95% CI, 1.38–2.80; *p* < 0.01) were independent prognostic factors for PFS (Table 3).

Therefore, Cox survival curves (OS and PFS) stratified by the CAR (≥ 0.30 vs. < 0.30) adjusting for these independent factors that showed significance on univariate analysis were made (Fig. 1e,f). The adjusted OS of patients with a CAR of ≥ 0.30 was significantly shorter than that of patients with a CAR of < 0.30 (adjusted HR, 4.79; 95% CI, 1.90–12.1; *p* < 0.01) (Fig. 1e). Similar results were also obtained for the adjusted PFS (adjusted HR, 3.82; 95% CI, 1.55–9.37; *p* < 0.01) (Fig. 1f).

### Comparison of the clinical characteristics and the outcomes according to the CAR

The clinical characteristics including the above factors related to OS or PFS on univariate analysis and the responses by the CAR were then compared (Table 4). The pretreatment CAR was lower than 0.30 in 25. The high-CAR group had poor ECOG PS, arterial infiltration, and high levels of NLR significantly (*p* < 0.01). Of these, ECOG PS and high levels of NLR were the significant predictors on univariate analysis for both OS and PFS (Table 3). Arterial infiltration was also a predictor of poor PFS and OS on univariate analysis, although PFS was marginally significant. In addition, patients with a high CAR showed a significantly lower disease control rate (PR + SD) than patients with a low CAR (7% vs. 68%, respectively) (Table 4).

**Table 4:** Comparison of the clinical characteristics and outcomes according to the CAR.

Items	CAR ≥ 0.30	CAR < 0.30	*P* value
	N = 21(%)	N = 25 (%)	
**Disease status**			0.81
Locoregional recurrence	11(52)	12(48)	
Distant recurrence	8(38)	9(36)	
Advanced at diagnosis	2(10)	4(16)	
**Measurable lesions**			0.48
Yes	14(67)	19(76)	
No	7(33)	6(24)	
**Arterial infiltration**			< 0.01
Yes	9(43)	1(4)	
No	12(57)	24(96)	
**ECOG PS**			< 0.01
0–1	10(48)	22(88)	
≥ 2	11(52)	3(12)	
**Nivolumab line**			0.8
1 or 2	15(71)	17(68)	
≥ 3	6(29)	8(32)	
**History of cetuximab-containing therapy**			0.98
Yes	11(52)	13(52)	
No	10(48)	12(48)	
**Radiation history**			0.87
Yes	18(86)	21(84)	
No	3(14)	4(16)	
**Distant metastases**			0.43
Yes	11(52)	16(64)	
No	10(48)	9(36)	
**NLR (**< **5 vs.** ≥ **5)**			< 0.01
< 5	5(24)	20(80)	
≥ 5	16(76)	5(20)	
**Best overall responses of patients with target lesions (N** = **33)**			
	N = 14	N = 19	< 0.01
PR	1(7)	4(21)	
SD	0(0)	9(47)	
PD	9(64)	5(26)	
NE	4(29)	1(5)	
ORR (%)	7	21	
DCR (%)	7	68	

### Comparison of the ROC curves between the CAR and the NLR

ROC curves for survival status at 2, 4, 6.3 (the median OS), and 8 months were constructed to compare AUC values to assess the discrimination ability between the CAR and the NLR (Fig. 2b–e). The CAR had significantly higher AUC values at 2 and 4 months compared with the NLR; the AUC values of CAR versus NLR were 0.96 versus 0.79 at 2 months (*p* = 0.02), and 0.83 versus 0.64 at 4 months (*p* < 0.01), respectively. In addition, the AUC values of CAR also tended to be higher than those of NLR at 6.3 months but not 8 months.

## Discussion

In this study, the pretreatment CAR was a more independent and significant prognostic factor in R/M HNSCC patients treated with nivolumab, compared with the NLR previously reported^10^. This result supports our expectation that the CAR is a novel prognostic indicator to predict both the prognosis of R/M HNSCC patients and the efficacy of nivolumab. To the best of our knowledge, this is the first report to investigate the prognostic usability of the CAR in nivolumab-treated R/M HNSCC patients.

A high pretreatment CAR was significantly associated with poor OS and PFS (Fig. 1c,d). A high level of the NLR (≥ 5), poor ECOG PS, and arterial infiltration were also significantly associated with poor OS. The NLR and poor ECOG PS were both previously reported to be significant prognostic factors for R/M HNSCC treated with nivolumab^10,21^. Notably, the high-CAR group was characterized by poor ECOG PS and arterial infiltration in the present study. This tendency might suggest that a high CAR reflects more advanced disease represented by cancer-induced cachexia^22,23^. In a retrospective study of patients with laryngeal squamous cell carcinoma, a higher CAR was shown to be associated with nodal metastasis and late-stage disease^22^. On the other hand, the modified Glasgow Prognostic Score (mGPS), a combination of albumin and the CRP level, was also reported to be associated with cancer cachexia and the prognosis in unresectable locally advanced head and neck cancer patients^23^. In addition, the CAR was found to be a more effective prognostic factor than ECOG PS or the NLR. Of note, comparison of the CAR with the NLR may be meaningful because the NLR was previously reported to be a biomarker for predicting the prognosis with nivolumab treatment for various types of cancer^7–9^ including R/M HNSCC^10^. In particular, the AUC values for the CAR at 2 and 4 months were shown to be significantly higher than the values for the NLR. The predictability was better with the CAR than with the NLR in terms of their usefulness to predict early prognosis, but not long-term outcome.

There is no consensus about the CAR cutoff for ICI-treated patients. Although the importance of CAR as a prognostic factor has already been reported in other squamous cell carcinoma (SCC) such as anal squamous cell carcinoma^24^ and esophageal cancer^25^, the proposed cutoff values vary in the several literatures. The major possible reasons would be the differences in the calculating method or patient background such as disease stage or cancer type and indeed, a positive correlation between disease stage and CAR has been reported in SCC^22,24^. While for preoperative head and neck cancer, the cutoff of CAR was set at 0.32^18^, it was set at 0.189 for cisplatin-based treated patients with metastatic nasopharyngeal carcinoma^26^. Therefore, it would be difficult to make a simple comparison with our results for the above reasons. On the other hand, Inoue et al. reported a cutoff value of 0.30 for the CAR in non-small cell lung cancer treated with nivolumab, and this cutoff value similarly reflected early prognosis^27^. This similarity might be noteworthy because of the same disease stage and method of calculating the cut off.

The CAR and NLR are certainly parallel markers in terms of indicators of inflammation, but the CAR reflects nutritional status. Therefore, it might be important to consider that this type of cancer expresses high levels of IL-6, inducing cachexia^28,29^. In fact, IL-6 signals inhibit several immunocompetent cells activation in the tumor microenvironment^30^. It also induces HNSCC cells to invade and metastasize^31^ and has been associated with recurrence and survival in HNSCC^30^. Locally or systemically elevated IL-6 levels have been reported to be associated with increased CRP concentrations in various cancers^32–35^. Furthermore, IL-6 also induces cachexia by altering the metabolism of lipids and proteins^36^. Recently, the correlation between the baseline IL-6 and CRP in melanoma treated with immune checkpoint inhibitors (ICIs) or chemotherapy was demonstrated, and higher levels of IL-6 were significantly associated with poor survival^37^. On the other hand, no significant change of systemic levels of IL-6 during ICI treatment has been observed in the studies of melanoma^38^ and gastric cancer^39^. In addition, several reports have already shown that CAR is an important prognostic factor for SCC treated with chemoradiotherapy^24^ or cytotoxic agents^26^. Therefore, CAR might not necessarily be an ICI-specific predictor, but a powerful prognosis factor for various types of cancer under different conditions. However, in a mouse model, the combined blockade of IL-6 and PD-1/PD-L1 signaling was shown to foster vigorous T-cell responses and decreased the cancer’s immunosuppressive activity^38^. It is an important clinical issue to determine whether we should avoid nivolumab use when the patients’ CAR score is high.

This study has several limitations. First, this was a retrospective study with a small sample size. In particular, the imaging test was conducted based on individual judgment. Therefore, PFS may not be a strict indicator of treatment efficacy. Second, the eligibility criteria were different from those of the CheckMate141 study on several points^3^. The study included 4 patients with different primary tumors from the CheckMate 141 study. In addition, 14 patients with poor PS (≥ 2) were also investigated. More importantly, one case had no history of platinum administration, and two received nivolumab as 1st-line therapy. Given such patient characteristics, it is not surprising that the median survival of 6.3 months in the present study was shorter than that of the CheckMate 141 study. In fact, since HNSCC patients with cancer-induced cachexia rarely met the eligibility criteria in the prospective clinical trial^23^, the present study would be significant as a report reflecting clinical practice. Despite the differences in background characteristics, the utility of the CAR was demonstrated by the adjusted analysis. It may be necessary to conduct a prospective clinical trial with a larger and more appropriate patient cohort to determine whether nivolumab would be the optimal option in patients with cachexia. To further elucidate the relationship between cachexia and cancer immunity, we plan to analyze the peripheral blood of cancer patients treated with ICI in a prospective cohort study.

In conclusion, the present study demonstrated that the pretreatment CAR was an independent marker of survival and efficacy of nivolumab in R/M HNSCC patients, and that the CAR was a better predictor than the NLR. The reason for the significant association between the CAR and nivolumab might be that patients with high CAR, which reflects not only inflammation but also more advanced stage like cachexia, already have some factors associated with poor prognosis. Furthermore, higher systemic inflammation at baseline might be the cause of the inhibitory effects on nivolumab-induced lymphocyte activation. From the perspective of molecular biology, further elucidation of the correlation between the CAR and serum cytokines and the immunological mechanism between inflammation and PD-1/PD-L1 signaling will be required.

## Methods

### Patients

The medical records of consecutive patients with R/M HNSCC who had been treated with nivolumab at two institutions from April 2014 to July 2019 were reviewed. This study was performed in line with the principles of the Declaration of Helsinki. Approval was granted by the Ethics Committee of Kyushu University Hospital (Approval No. 2019-573). Because of the retrospective nature of the present study, informed consent was not obtained from each patient. The consent was waived by the Ethics Committee of Kyushu University Hospital. The eligibility criteria were: (1) histologically confirmed R/M HNSCC; (2) no previous immunotherapy; and (3) older than 20 years of age. This study did not place restrictions on platinum use history, treatment lines, and ECOG PS.

### Treatment evaluation

The pretreatment baseline characteristics of each patient were retrospectively examined using electronic records. Items surveyed in this study included age, sex, ECOG PS, primary tumor site, Histological differentiation, p16 status, line of nivolumab administration, body mass index (BMI) (kg/m^2^), CRP (mg/dl), serum albumin (g/dl), CAR, NLR, history of previous therapy and disease status (locoregional recurrence, distant recurrence, or advanced at diagnosis). To determine the presence of arterial infiltration, we referred CT or MRI reports, and defined the positive arterial infiltration based on the encasement of the half circumference or deformation of the artery by the tumor on the images. Information about efficacy included best overall response, PFS, OS, and reasons for nivolumab termination. Assessment of tumor lesions was performed by CT every 2–3 months. In cases of worsening subjective symptoms or laboratory findings, CT was performed. Target lesion and best overall response were assessed according to the Response Evaluation Criteria in Solid Tumors (RECIST), version 1.1^40^. The CAR was calculated by dividing the serum CRP by the serum albumin. The NLR was derived from the absolute neutrophil and absolute lymphocyte counts of a full blood count. In the present study, an NLR of 5 was used as the threshold value, since its clinical utility in predicting patient outcomes in a variety of cancers was examined in previous reports^8,10^.

### Treatment exposure

Nivolumab was administered at a dose of 3 mg/kg or 240 mg/body every 2 weeks. This regimen was repeated until disease progression, death, unacceptable toxicities, or patient refusal.

### Cutoff value for the CAR

The cutoff value for the CAR was identified by ROC curve analysis. Outcome prediction at the median survival time was used as the state variable. The optimal CAR threshold, which corresponded to the maximum sum of sensitivity and specificity on the ROC curve, was determined. To evaluate the discriminatory ability of the CAR and the NLR, ROC curves were generated, and differences between the AUCs were compared using the method established by DeLong et al.^41^.

### Statistical analysis

PFS was defined as the period from the initiation of nivolumab to the date of tumor progression determined by each physician or death from any cause, whichever was earlier, or was censored at the final follow-up. OS was defined as the period from initiation of nivolumab to the date of death from any cause or was censored at the final follow-up. PFS and OS were estimated using the Kaplan–Meier method, and the log-rank test was used to compare survival curves between the two groups using the cutoff value for the CAR.

Univariate and multivariate analyses for OS and PFS were conducted with the Cox proportional hazards model. Multivariate Cox hazard regression analysis was performed with variables that showed significance on univariate analysis. HRs and corresponding 95% CIs are reported. In the univariate and multivariate analyses, the CAR was analyzed as a continuous variable.

To assess the correlations between the CAR and other factors, patients were stratified into two groups by different factors. Comparisons of clinical characteristics of these groups were conducted using the Pearson Chi-Squared test. Values of *p* < 0.05 were considered significant. All statistical analyses were performed using JMP, version 14 (SAS Institute, Cary, NC) and R, version 3.6.1 (R Foundation for Statistical Computing, Vienna, Austria).

## Data Availability

The datasets generated during the current study are available from the corresponding author on reasonable request.
